# Pediatric In‐Hospital Cardiac Arrest in Sweden: A Tertiary Care Cohort With High Survival

**DOI:** 10.1111/aas.70302

**Published:** 2026-07-12

**Authors:** Hannah Fovaeus, Johan Holmen, Zacharias Mandalenakis, Matilda Frisk Torell, Araz Rawshani, Albert Gyllencreutz Castellheim

**Affiliations:** ^1^ Department of Anesthesiology and Intensive Care Medicine, Institute of Clinical Sciences, Sahlgrenska Academy University of Gothenburg Gothenburg Sweden; ^2^ Department of Pediatrics Sahlgrenska University Hospital Gothenburg Sweden; ^3^ Department of Pediatric Anesthesiology and Intensive Care Sahlgrenska University Hospital Gothenburg Sweden; ^4^ Department of Molecular and Clinical Medicine, Sahlgrenska Academy University of Gothenburg Gothenburg Sweden; ^5^ Department of Medicine, Adult Congenital Unit Sahlgrenska University Hospital Gothenburg Sweden; ^6^ Department of Cardiology Sahlgrenska University Hospital Gothenburg Sweden

## Abstract

**Background:**

Pediatric in‐hospital cardiac arrest is a rare event. Despite Sweden's extensive national health registries, data describing pediatric in‐hospital cardiac arrest are limited. In 2018, pediatric‐specific variables were introduced into the Swedish Registry for Cardiopulmonary Resuscitation. This study aimed to identify the characteristics and outcomes of pediatric IHCA at a Swedish tertiary pediatric referral hospital following implementation of these variables.

**Methods:**

This observational cohort study included all pediatric in‐hospital cardiac arrest events with initiated cardiopulmonary resuscitation (CPR) recorded at Queen Silvia Children's Hospital between 2018 and 2025. Demographic characteristics, arrest location, resuscitation variables, and survival outcomes were analyzed. Survival was assessed using Kaplan–Meier methods and Cox proportional hazard regression.

**Results:**

A total of 125 in‐hospital cardiac arrest events were included. Infants (< 1 year) represented 65.6% of cases and 57% of patients had congenital heart disease. Most arrests occurred in the intensive care unit (56.8%), followed by hospital wards (21.6%) and the operating room (16.0%). CPR was initiated within the first minute in 81.3% of cases. Return of spontaneous circulation was achieved in 90% of patients. Survival was 82% at 30 days and 68% at 1 year.

**Conclusions:**

Pediatric in‐hospital cardiac arrest occurring in highly monitored tertiary care environments has a very high rate of return of spontaneous circulation as well as survival. This study also highlights the importance of systematic registry data collection to improve understanding of pediatric in‐hospital cardiac arrest and to support future benchmarking and quality improvement.

**Editorial Comment:**

In‐hospital cardiac arrest is rare for pediatric patients. This retrospective cohort analysis, from one tertiary care pediatric hospital which includes a large pediatric cardiac surgery activity, presents pediatric cardiac arrest events in the hospital, along with case factors.

## 1 Introduction

Pediatric in‐hospital cardiac arrest (IHCA) is a rare but life‐threatening event [1]. Despite Sweden's long tradition of high‐quality national health registries [2], national data describing pediatric IHCAs are lacking. This knowledge gap is not unique to Sweden. Across Europe, only eight countries report national registry data on pediatric IHCA [3]. In contrast, 11 countries report adult IHCA with registry coverage exceeding 80% [4].

The reported incidence of adult IHCA in Europe ranges from 1.5 to 2.8 per 1000 hospital admissions [4], with an incidence of approximately 1.7 per 1000 admissions reported in Sweden [5]. The Swedish Registry for Cardiopulmonary Resuscitation (SRCR) is a national quality registry that has collected data on IHCAs since 2005 and covers the vast majority of cases nationwide [6]. However, large pediatric hospitals and departments have historically not participated in the registry, as the recorded variables primarily reflected characteristics of adult cardiac arrest and cardiopulmonary resuscitation (CPR). Pediatric‐specific variables were introduced in the registry in 2018.

National cardiac arrest registries provide important opportunities for benchmarking and quality improvement, and evaluation of resuscitation practices. Previous studies have demonstrated that benchmarking models can contribute to reductions in morbidity and mortality [7, 8]. The absence of comprehensive pediatric IHCA data limits opportunities for structured quality improvement and outcome comparisons.

Survival following pediatric IHCA shows considerable variability across studies and settings [1, 9]. Return of spontaneous circulation (ROSC) after cardiac arrest with CPR may reach 80%–90%, whereas survival to hospital discharge is often reported to be below 50% [10]. Most pediatric cardiac arrests occur in intensive care units (ICUs), cardiac ICUs, or other monitored settings [11]. This may be advantageous compared with cardiac arrests occurring in a general ward. Previous studies have reported that 2%–6% of pediatric ICU admissions involve cardiac arrest and that up to 93% of cardiac arrests occur in the ICU [12]. Factors associated with improved survival include elective admissions, treatment in teaching hospitals, and weekday admissions [13]. Whether these patterns are applicable to the Swedish pediatric in‐hospital population remains unknown.

The aim of this study was to identify the characteristics and outcomes of pediatric IHCA at a Swedish tertiary pediatric center following the implementation of pediatric‐specific variables in the SRCR.

## 2 Methods

### 2.1 Study Design

This observational cohort study was based on data from the SRCR. Data collection in the SRCR follows the Utstein reporting template for cardiac arrest research [14].

### 2.2 The SRCR

The SRCR was established in 1990 for out‐of‐hospital cardiac arrests (OHCA) and expanded in 2005 to include IHCAs. The registry records cardiac arrest events in which CPR was initiated. In 2018, pediatric‐specific variables were introduced, enabling more accurate registration and analysis of pediatric cardiac arrest events. For example, that asystole/bradycardia in small children can be equivalent to a heart rhythm of less than 60 beats per minute or emphasize the pediatric etiologies such as respiratory failure and severe hypovolemia.

Multiple cardiac arrest events can be recorded for the same individual, although only one event per patient per day can be registered. For the present study, only the first cardiac arrest event per person was included.

Data entry into the SRCR is performed digitally. Ideally, the first part of the registration is completed by the healthcare professionals present at the cardiac arrest event. Time‐critical variables—including time of cardiac arrest, of alarm, of initiation of CPR, and of ROSC—are documented as close to the event time as possible. The second part of the registration is completed retrospectively by trained personnel who review the medical records to register background characteristics and detailed information regarding the cardiac arrest and resuscitation efforts.

### 2.3 Setting

Queen Silvia Children's Hospital is a tertiary pediatric referral center, teaching hospital, and a community hospital with approximately 9000 pediatric hospital admissions annually. Age‐stratified admission data were not available. The hospital serves as a national referral center for congenital heart disease for the northern half of Sweden and performs approximately 250 pediatric cardiac surgical procedures per year.

Due to the hospital's specialized congenital heart disease services, the pediatric ICU manages a large number of cardiac patients, both in association with cardiac surgery and other cardiac conditions. Since the introduction of pediatric variables in the SRCR in 2018, the hospital has prioritized comprehensive reporting of IHCA events with CPR, including cases occurring in the ICU and operating room (OR).

### 2.4 Study Population and Dataset

In total 125 pediatric IHCA events in which CPR was initiated were included. All of them were at Queen Silvia Children's Hospital between 2018 and 2025. The dataset comprised demographic, clinical, event‐related, and outcome variables.

Demographic variables included age at arrest, sex, congenital heart disease status, and prematurity. Event characteristics included location of arrest, defibrillation status, and time intervals related to the resuscitation process. Outcomes included ROSC, 30‐day survival, and 1‐year survival.

### 2.5 Statistical Analyses

Statistical analyses were performed using SAS software (version 9.4). Continuous variables are presented as medians with interquartile ranges (IQR), and categorical variables as counts and percentages.

Survival analyses were performed using Kaplan–Meier curves stratified by age, sex, and location of arrest, with log‐rank tests used for univariable comparisons. Multivariable survival analyses were performed using Cox proportional hazard regression. Hazard ratios (HRs) with 95% confidence intervals (CIs) were calculated for both unadjusted models and models adjusted for age and location of arrest. Because of the small number of patients in older age groups, some age categories were combined in the Kaplan–Meier figure for visual clarity. The Cox regression analyses used the prespecified age groups shown in Table 3.

Incidence rates were calculated as events per 10 person‐years with 95% CIs. Statistical significance was defined as *p* < 0.05 (two sided).

The Cox model was assessed for the proportional hazard assumption using Schoenfeld residuals. Model predictive performance was evaluated using Uno's *C*‐index.

## 3 Results

### 3.1 Study Population

A total of 125 IHCAs were included during the study period. The incidence was 2.3 cardiac arrests per 1000 hospital admissions. Most arrests occurred in infants (< 1 year of age), representing 65.6% of cases (*n* = 82). The median age at arrest was 0 years (range 0–17). A majority, 60% of patients were male (*n* = 75) and 40% female (*n* = 50). Congenital heart disease was present in 57.0% (*n* = 69) of patients and 31.4% (*n* = 33) had a history of preterm birth.

### 3.2 Location and Arrest Characteristics

Most cardiac arrests occurred in the ICU (56.8%, *n* = 71). Additional cardiac arrests also occurred in hospital wards (21.6%, *n* = 27), the OR (16.0%, *n* = 20), and the emergency department (3.2%, *n* = 4) excluding patients arriving with ongoing cardiac arrest. Defibrillation was required in seven cases (5.6%), while the majority of arrests (88.0%, *n* = 110) were managed without defibrillation.

### 3.3 Resuscitation Process

Median time from cardiac arrest to initiation of CPR was 0 min (mean 0.48 min, SD 1.54; *n* = 123) with immediate CPR in 81.3% of cases. Median time from arrest to alarm activation was 0 min (mean 0.42 min, SD 0.74; *n* = 41), and median time to resuscitation team arrival was 3 min (mean 2.85 min, SD 2.40; *n* = 34).

ROSC occurred after a median of 3 min (mean 8.12, SD 15.63; *n* = 95) (Table 1).

**TABLE 1:** Demographics, clinical characteristics, and response times.

Variable	All (*n* = 125)
Demographics and clinical characteristics	
Age group, *n* (%)	
0 years	82 (65.6%)
1–5 years	26 (20.8%)
6–10 years	7 (5.6%)
11–15 years	6 (4.8%)
16–18 years	4 (3.2%)
Age at cardiac arrest, median (IQR), years	0 (0; 17)
Congenital heart disease, *n* (%)	69 (57.0%)
Prematurity, *n* (%)	33 (31.4%)
Female, *n* (%)	50 (40%)
Arrest characteristics	
Location of cardiac arrest, *n* (%)	*n* = 125
Ward	27 (21.6%)
ICU	71 (56.8%)
Emergency department	4 (3.2%)
Operating room	20 (16.0%)
Other	3 (2.4%)
Defibrillation performed, *n* (%)	*n* = 125
No	110 (88.0%)
Yes	7 (5.6%)
Missing	8 (6.4%)
Response times	
Arrest to alarm, median (IQR), min	0 (0; 1)
(categorical), *n* (%)	*n* = 48
< 0	7 (14.6%)
0	29 (60.4%)
1–5	8 (16.7%)
6–10	3 (6.3%)
≥ 11	1 (2.1%)
Arrest to CPR initiation, median (IQR), min	0 (0; 0)
(categorical), *n* (%)	n = 123
0	100 (81.3%)
1–5	21 (17.1%)
6–10	1 (0.8%)
≥ 11	1 (0.8%)
Arrest to team arrival, median (IQR), min	3 (0; 5)
(categorical), *n* (%)	*n* = 34
< 0	6 (15.0%)
0	9 (22.5%)
1–5	21 (52.5%)
6–10	4 (10.0%)
≥ 11	0 (0.0%)
Arrest to ROSC, median (IQR), min	3 (1; 8)
(categorical), *n* (%)	*n* = 95
0	6 (6.3%)
1–5	58 (61.1%)
6–10	14 (14.7%)
≥ 11	17 (17.9%)

Abbreviations: CPR, cardiopulmonary resuscitation; IQR, interquartile range; ROSC, return of spontaneous circulation.

### 3.4 Survival

Overall, 90% of patients achieved ROSC. Thirty‐day survival was 82% and 68% were alive after 1 year (Table 2). Among patients requiring defibrillation (*n* = 7), ROSC was achieved in 71% (*n* = 5). Thirty‐day and 1‐year survival in this group were both 43% (*n* = 3).

**TABLE 2:** Return of spontaneous circulation and survival after 30 days and 1 year after in‐hospital cardiac arrest at Queen Silvia Children's hospital.

Variable		ROSC	30 days	1 year
Total		0.90	0.82	0.68
Sex	Male	0.91	0.81	0.68
	Female	0.90	0.84	0.68
Age (year)	0	0.96	0.90	0.72
	1–5	0.77	0.69	0.62
	6–10	0.71	0.43	0.43
	11–15	1.00	0.83	0.83
	16–18	0.75	0.75	0.50
Defibrillation	No	0.92	0.85	0.71
	Yes	0.71	0.43	0.43
Location	Ward	0.85	0.81	0.67
	Intensive care	0.93	0.86	0.72
	Emergency dep.	0.75	0.75	0.50
	Operating room	0.90	0.70	0.55
	Other	1.00	1.00	1.00

**FIGURE 1:**
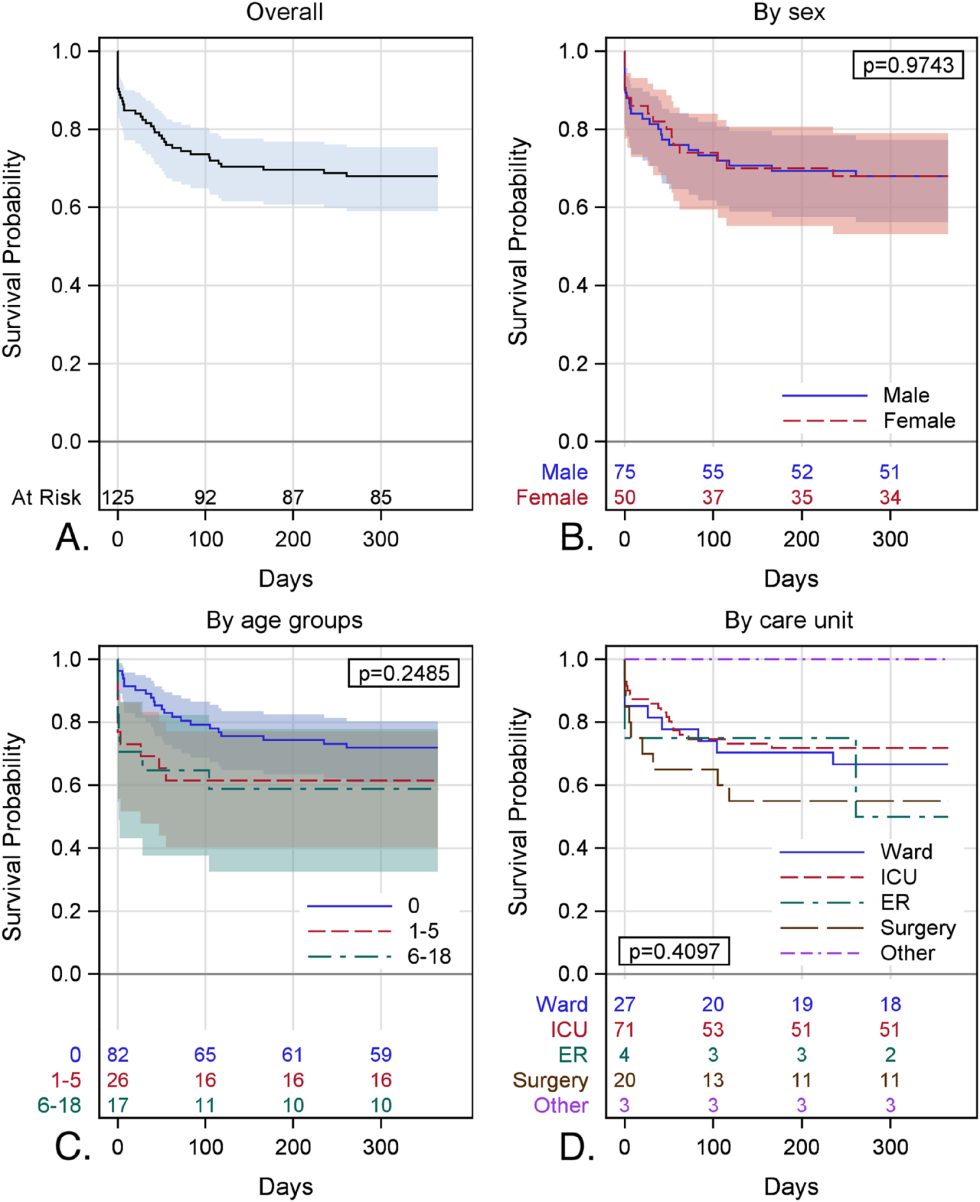
One‐year survival demonstrated with Kaplan–Meier curves. (A) overall survival, (B) survival based on sex, (C) survival based on age in years, and (D) survival based on arrest location.

No statistically significant differences in survival were observed between males and females (*p* > 0.05). Survival also did not differ significantly according to arrest location or age groups (Figure 1).

### 3.5 Factors Associated With Survival

Despite the lack of statistically significant differences across age groups overall, survival varied substantially between specific groups. Survival was 90% among infants (< 1 year) compared with 43% in children aged 6–10 years (Table 2).

In adjusted Cox regression analysis, children 6–10 years had a higher mortality risk compared with infants but did not reach statistical significance (adjusted HR 7.33, 95% CI 2.09–25.68, *p* = 0.0018) (Table 3).

**TABLE 3:** Hazard ratio 1‐year.

					Unadjusted			Adjusted		
Value	Events/*N* (%)	Total time (years)	Events per 10 years (95% CI)	Log‐rank *p*‐value	HR (95% CI)	Cox *p*‐value	Uno *C*‐index	Adjusted HR (95% CI)	Cox *p*‐value	Uno *C*‐index
Sex										
Male (ref.)	24/75 (32.0%)	53.8	4.46 (2.97; 6.70)			0.97**			0.91**	
Female	16/50 (32.0%)	36.0	4.44 (2.69; 7.34)	0.9743	0.99 (0.53; 1.86)	0.97	0.50	0.96 (0.50; 1.87)	0.91	0.62
Age										
0 (ref.)	23/82 (28.0%)	63.1	3.64 (2.41; 5.52)			0.18**			0.24**	
1–5	10/26 (38.5%)	16.4	6.12 (3.19; 11.73)		1.62 (0.77; 3.42)	0.20		1.73 (0.81; 3.70)	0.15	
6–10	4/7 (57.1%)	3.0	13.30 (3.91; 45.21)		3.15 (1.08; 9.15)	0.035		2.94 (0.98; 8.80)	0.054	
11–15	1/6 (16.7%)	5.1	1.97 (0.15; 25.77)		0.58 (0.08; 4.28)	0.59		0.58 (0.08; 4.38)	0.60	
16–18	2/4 (50.0%)	2.3	8.76 (0.92; 83.11)	0.0402	2.08 (0.49; 8.81)	0.32	0.60	1.94 (0.45; 8.38)	0.38	0.65
Location										
Ward	9/27 (33.3%)	19.3	4.66 (2.35; 9.24)			0.64**			0.69**	
Intensive care	20/71 (28.2%)	53.0	3.77 (2.42; 5.89)		0.82 (0.37; 1.80)	0.62		0.81 (0.37; 1.79)	0.61	
Emergency dep.	2/4 (50.0%)	2.7	7.37 (0.78; 69.95)		1.55 (0.33; 7.17)	0.58		1.65 (0.35; 7.68)	0.52	
Operating room	9/20 (45.0%)	11.8	7.64 (3.80; 15.35)		1.49 (0.59; 3.74)	0.40		1.38 (0.54; 3.51)	0.50	
Other	0/3 (0.0%)	3.0	0.00 (0.00)	0.4097	0.00 (0.00)	0.99	0.57	0.00 (0.00)	0.99	0.62
Defibrillation										
No (ref.)	32/110 (29.1%)	82.1	3.90 (2.75; 5.53)			0.097**			0.20**	
Yes	4/7 (57.1%)	3.1	12.98 (3.82; 44.12)		2.77 (0.98; 7.87)	0.055		2.36 (0.66; 8.47)	0.19	
Missing	4/8 (50.0%)	4.6	8.62 (2.64; 28.13)	0.0748	1.90 (0.67; 5.37)	0.23	0.56	2.03 (0.65; 6.31)	0.22	0.65

*Note:* Adjusted Cox regression analyses, adjusted for age and place of arrest. **p‐value for the entire effect/factor/variable.

## 4 Discussion

To our knowledge, this study provides the first detailed description of pediatric IHCA in Sweden following the introduction of pediatric‐specific variables in the SRCR. The study presents a 6‐year single‐center experience (2018–2025) from a tertiary pediatric referral hospital. Although the sample size is limited, the findings provide important insights into the characteristics, management, and outcomes of pediatric IHCA in a Swedish hospital setting.

Three observations from this study deserve particular attention. First, the rate of ROSC was very high, occurring in 90% of patients. This proportion exceeds other reported pediatric IHCA cohorts [3]. The high ROSC rate in this cohort should be interpreted in the context of the study setting. Many events occurred in highly monitored environments, such as the ICU and OR. Here, cardiac arrest may be preceded by observed physiological deterioration, and CPR may be started early. These events may differ from sudden cardiac arrests in less monitored hospital areas. This is important when comparing our results with other pediatric IHCA cohorts and when considering generalizability. This interpretation is supported by the short response times observed, with CPR initiation within the first minute in 81.3% of cases. This is consistent with previous studies reporting IHCA [12]. In some studies, CPR events lasting less than 1 min are excluded because of uncertainty regarding whether a true cardiac arrest occurred [15]. In the SRCR, however, initiation of CPR is defined as either chest compressions or defibrillation [16]. We considered these events clinically relevant, as the outcome might have been different had resuscitation not been initiated. In our cohort, 67% of patients achieved ROSC within 5 min, further supporting that rapid recognition and initiation of CPR occurred. Consequently, some response variables such as time from arrest to alarm and time to team arrival were missing in a number of cases, as emergency teams were not always activated in the ICU or OR (Table 1).

Second, survival in this cohort was also high. Thirty‐day survival was 82% and 1‐year survival was 68%, which compare favorably with previously reported pediatric IHCA populations [10]. The decrease in survival between ROSC and longer‐term follow‐up likely reflects the substantial underlying disease burden among children treated at this center, particularly the high prevalence of congenital heart disease [15]. The high proportion of patients with congenital heart disease likely influenced the age distribution and survival patterns observed. In contrast, pediatric cardiac arrests caused by conditions such as septic shock are known to be associated with particularly poor outcomes [17].

Third, infants (< 1 year) represented nearly two‐thirds of all cardiac arrests in this cohort. This differs from several pediatric IHCA studies in which age distribution is more evenly spread across childhood [10]. We could not calculate an age specific incidence rate for infant admissions. This limits interpretation of whether infants had a higher risk of IHCA compared with older children. The predominance of infants and patients with congenital heart disease likely reflects the tertiary referral profile of the hospital. The relatively high degree of prematurity, over 30% in this study compared with approximately 5% in a general population, demonstrates the association between congenital heart disease and premature birth [18, 19].

Only seven patients required defibrillation in our study, and survival in this group was relatively low. Given the very small sample size, these findings should be interpreted with caution and may primarily reflect the underlying severity of illness in these individual cases rather than shockable rhythm being a negative prognostic value [20].

Prevention is a major focus in pediatric cardiac arrest research. Many hospitals have implemented medical emergency teams or rapid response systems, where ICU‐trained staff assess patients showing early signs of deterioration on hospital wards [21]. These interventions aim to prevent cardiac arrest by initiating earlier treatment or transferring patients to higher levels of care [9]. Emerging approaches also include the use of machine learning models to predict cardiac arrest several hours before the event in pediatric intensive care settings [22]. While these technologies may offer promising opportunities, their implementation requires careful evaluation to ensure that predictive models are reliable and clinically meaningful [23].

We did not find statistically significant differences in survival between age groups, sex, or arrest location. Children aged 6–10 years had a lower observed survival than infants, and the adjusted HR was higher than 1, but this result did not reach statistical significance. This finding should therefore be interpreted with caution. The number of children in this age group was small, and the study was not powered to detect moderate differences between subgroups. The observed difference may reflect variation in underlying disease, cause of arrest, or care setting, but we could not study etiological subgroups in this dataset. This is an important limitation.

Several limitations should be considered. The study was conducted at a single tertiary referral center, which may limit generalizability. The sample size was relatively small, restricting statistical power for subgroup analyses. In addition, the analysis was limited to variables available within the SRCR, and detailed information regarding etiology and post‐resuscitation management was not available. Capturing cardiac arrest events, especially in monitored settings, ICU and ORs, may be challenging for registry‐based research, as alarms are not always activated and the events may also be more difficult to identify retrospectively. Nevertheless, our study included 20 perioperative cardiac arrests in the OR [24].

Because pediatric IHCA is rare, systematic data collection is essential for benchmarking and quality improvement. Comprehensive reporting of cardiac arrest events across all hospital settings, including ICUs and ORs, will be important for enabling meaningful comparisons between centers [25]. Such data will enable improved benchmarking, identification of modifiable risk factors, and the development of targeted quality improvement initiatives. Future studies should link registry data with clinical monitoring data or electronic health records to describe pre‐arrest physiology and common pathways to pediatric IHCA. Expansion of pediatric data within the SRCR will allow for future national analyses of pediatric IHCA in Sweden and may help identify modifiable risk factors and guide quality improvement initiatives. Future studies should also focus on the underlying etiologies of pediatric cardiac arrest and long‐term neurological outcomes.

## 5 Limitations

Several limitations should be considered. First, this was a single center study from a tertiary pediatric referral hospital, and many events occurred in highly monitored settings such as the ICU and OR. The high ROSC rate may therefore partly reflect early recognition of physiological deterioration and rapid initiation of CPR, rather than outcomes after sudden unwitnessed cardiac arrest. Second, the SRCR does not include detailed pre‐arrest physiological data, such as heart rate, thus not describing the clinical trajectory parameters in the hour before the event. We could therefore not describe the clinical trajectories leading to arrest. Third, the registry definition is based on initiation of CPR, defined as chest compressions or defibrillation. In pediatric intensive care settings, this may include peri‐arrest or impending arrest events, especially severe bradycardia with rapid clinical deterioration. This may affect the reported ROSC rate and should be considered when comparing our results with other studies.

## 6 Conclusion

Pediatric IHCA occurring in highly monitored tertiary care environments has a very high rate of ROSC as well as survival. This study also highlights the importance of systematic registry data collection to improve understanding of pediatric IHCA and to support future benchmarking and quality improvement.

## Author Contributions

**Hannah Fovaeus:** conceptualization, data curation, formal analysis, investigation, methodology, writing – original draft, writing – reviewing and editing. **Johan Holmen:** conceptualization, investigation, methodology, writing – reviewing and editing. **Zacharias Mandalenakis:** methodology, writing – reviewing and editing. **Matilda Frisk Torell:** conceptualization, methodology, writing – reviewing and editing. **Araz Rawshani:** conceptualization, methodology, writing – reviewing and editing. **Albert Gyllencreutz Castellheim:** conceptualization, data curation, formal analysis, methodology, supervision, writing – reviewing and editing.

## Funding

The authors have nothing to report.

## Ethics Statement

The ethical committee in Lund approved the ethical permission for the present analysis September 30, 2025 (Dnr 2025‐05759‐01).

## Conflicts of Interest

The authors declare no conflicts of interest.

## Data Availability

The data that support the findings of this study are available on request from the corresponding author. The data are not publicly available due to privacy or ethical restrictions.
